# Cervical Carcinogenesis and Immune Response Gene Polymorphisms: A Review

**DOI:** 10.1155/2017/8913860

**Published:** 2017-02-09

**Authors:** Akash M. Mehta, Merel Mooij, Ivan Branković, Sander Ouburg, Servaas A. Morré, Ekaterina S. Jordanova

**Affiliations:** ^1^Department of Pathology, Leiden University Medical Centre, Leiden, Netherlands; ^2^Laboratory of Immunogenetics, Department of Medical Microbiology and Infection Control, VU University Medical Centre, Amsterdam, Netherlands; ^3^Institute for Public Health Genomics, Department of Genetics and Cell Biology, School for Oncology and Developmental Biology (GROW), Faculty of Health, Medicine and Life Sciences, Maastricht University, Maastricht, Netherlands; ^4^Centre for Gynaecological Oncology Amsterdam, Amsterdam, Netherlands

## Abstract

The local immune response is considered a key determinant in cervical carcinogenesis after persistent infection with oncogenic, high-risk human papillomavirus (HPV) infections. Genetic variation in various immune response genes has been shown to influence risk of developing cervical cancer, as well as progression and survival among cervical cancer patients. We reviewed the literature on associations of immunogenetic single nucleotide polymorphism, allele, genotype, and haplotype distributions with risk and progression of cervical cancer. Studies on HLA and KIR gene polymorphisms were excluded due to the abundance on literature on that subject. We show that multiple genes and loci are associated with variation in risk of cervical cancer. Rather than one single gene being responsible for cervical carcinogenesis, we postulate that variations in the different immune response genes lead to subtle differences in the effectiveness of the antiviral and antitumour immune responses, ultimately leading to differences in risk of developing cervical cancer and progressive disease after HPV infection.

## 1. Introduction

Infection with human papillomavirus (HPV) is highly common across human populations. Worldwide, prevalence estimates of HPV infection among women range from 2% to 44% [1]. Invasive cervical carcinoma, which is caused by malignant transformation of cervical epithelial cells following persistent HPV infection, is one of the most common malignant diseases among women, representing almost 10% of all cancers in the female population. Each year, more than 500.000 women are diagnosed with cervical cancer, mostly in developing countries [2].

Approximately 200 HPV types have been identified to date, with new types constantly being discovered. Types may differ in tissue tropism and may preferentially infect skin or mucosa. Certain HPV types are pathogenic, leading to a variety of benign conditions (genital, oral, and throat warts) as well as malignant disease (the most common being cervical, penile, vulvar, vaginal, and oesophageal carcinoma) [3, 4]. HPV types 16 and 18 are responsible for approximately 60–80% of all cervical cancer cases, while types 52 and 31 account for the majority of the remaining cases. However, HPV distribution patterns differ significantly amongst various populations [5].

Although infection and colonisation of the cervical epithelium by oncogenic, high-risk human papilloma viruses (hr-HPVs) are prerequisites for the development of cervical cancer, the local immune response is thought to be an important determinant of progression and disease outcome [6]. The higher incidence of HPV-associated cervical dysplasia in immunosuppressed patients supports the hypothesis that local immune responses are an important determinant in transformation of epithelial cells [6]. The transiency of most HPV infections and the observed regression of certain cervical intraepithelial neoplasia (CIN) lesions to normal epithelium suggest a variability in local immune responses, which may be caused by differences in host genomics [7].

Genetic variation in various immune mediators has been shown to be an important determinant in susceptibility to a wide variety of autoimmune disorders and neoplasms, as well as in progression and disease outcome [8–10]. This is especially the case for HPV-related epithelial transformation [11]. Understanding immunogenetic variation is necessary not only to comprehend the striking heterogeneity in anti-HPV and antitumour immune responses but also to enable and facilitate rational design of host-directed therapy and other novel treatment modalities. This review aims to provide an overview of common single nucleotide polymorphisms (SNPs) in genes encoding cytokines, chemokines, receptors, and antigen-processing machinery (APM) components and association with cervical carcinoma risk, progression, and/or outcome.

## 2. Methods

A systematic search in the NCBI PubMed bibliographic database and HuGE navigator was conducted [46]. Since major histocompatibility complex (MHC/HLA) and killer cell immunoglobulin-like receptor (KIR) genes have been abundantly studied in cervical cancer [47–55], these terms were excluded from the search. All original research studies and meta-analyses, published until August 1st 2015 and reporting on genes encoding any immune response mediators and either risk of cervical cancer or survival outcome amongst cervical cancer patients, were included.

Information on allele or genotype frequencies and, if available, odds or hazard ratios with associated 95% confidence intervals were extracted from the studies. If unavailable in the original studies, these ratios were calculated along with the population attributable faction (PAF), according to Miettinen's formula [56].

## 3. Cytokines

Cytokines play a crucial role in mounting and maintaining immune responses against a host of pathogens, including viral infections and tumours [11]. Though many different classification systems exist for these signalling molecules, the most basic subdivision is that of proinflammatory and anti-inflammatory cytokines. A separate group is formed by chemokines, chemoattractant cytokines specifically involved in chemotaxis, the process by which different immune cells are recruited and signaled to migrate to certain sites to build a local inflammatory response. Among the most ubiquitous cytokines are the interleukins, which influence development and differentiation of lymphocytes and hematopoietic cells. Cytokines are produced by many different cells involved in the immune response; substantial evidence suggests that certain tumour cells, both in vitro and in vivo, also generate various cytokines [57, 58]. Genetic variation in genes encoding proinflammatory (Table 1) and anti-inflammatory cytokines (Table 2) and chemokines (Table 3) may lead to altered function or quantity of the associated cytokines and is therefore believed to be an important determinant of anti-HPV and antitumour immunity.

### 3.1. Proinflammatory Cytokines

Although several polymorphisms in the genes encoding various interleukins have been described in relation to cervical carcinoma and its precursor lesions [59–63], the most consistently reported SNP is in the promotor region of the* IL1B* gene (c.-511C>T) encoding interleukin-1 beta (mediator of the inflammatory response, cell proliferation, differentiation, and apoptosis), which has been demonstrated to be associated with cervical carcinoma risk in Korean, North Indian, Chinese Han, and Egyptian populations [12–15]. Two recent meta-analyses demonstrated significant association of the minor allele with increased cervical cancer risk [16, 64].

Several polymorphisms in the* TNF* gene, encoding tumour necrosis factor (TNF), have been described in relation to HPV infection and cervical neoplasia. TNF is a key proapoptotic cytokine, involved in cell proliferation and differentiation [65]. Variation at one site in the promotor region (c.-308G>A) has been found to be associated with development of CIN lesions and with susceptibility to HPV16 infection and subsequent cervical carcinoma in various populations. It has been hypothesised that the minor allele is associated with increased TNF production, which may be associated with induction of angiogenesis, a prerequisite for cancer cell growth and progression [17]. However, the genotypes associated with HPV-related epithelial transformation differed among the investigated populations: in a British cohort, all categories of CIN were associated with major allele homozygosity, while in Indo-Aryan and Portuguese women, the minor allele was associated with a threefold increase in susceptibility to cervical cancer and a twofold increased risk of developing cervical cancer in Portuguese women [17, 18, 66]. A recent meta-analysis demonstrated association of the minor allele with cervical cancer risk (OR 1.19; 95% CI 1.02–1.38), although there was significant population-based heterogeneity [67]. Data regarding other* TNF* gene polymorphisms, including microsatellite polymorphisms, is available but conflicting, probably due to small sample sizes [68–70].

Interferon-gamma (IFN*γ*) plays an important role in antiviral immunity [71]. Genetic variation of the c.874T>A locus, located at the translation start site of the first intron of the* IFNG *gene, has been shown to be associated with altered levels of IFN*γ* production in response to immunogenic stimuli, with minor allele carriers having decreased IFN*γ* production [72]. In several North Indian cohorts, genotypes with the minor allele were associated with increased risk and with higher disease stage [19, 20]. Minor allele homozygosity was also associated with increased cervical cancer risk in a Chinese population [21]. However, studies among Brazilian and Swedish populations did not find any significant associations of this polymorphism with cervical carcinoma [73–75]. Two recent meta-analyses have demonstrated a clear association of the minor allele at this locus with increased cervical carcinoma risk; this association is the strongest among Asian populations [16, 22]. Possible explanations for this difference among populations could include differences in HPV type distributions as well as in background host genomics.

### 3.2. Anti-Inflammatory Cytokines

The* IL1RN* gene encodes the interleukin-1 receptor antagonist (IL-1RA), which regulates the biological activity of the two potent proinflammatory cytokines interleukin-1*α* and interleukin-1*β* [76]. Most of the genetic variation in this gene is attributed to a polymorphic 86-bp variable numbers tandem repeat (VNTR) in intron 2; five different allelic variants of this VNTR have been described. Two studies among Indian populations showed association of genetic variation at this site with cervical carcinoma risk, with allele 2 bearing the strongest association with increased cancer risk [13, 77], both in single genotype analysis and in haplotype analysis with the* IL1B* gene. In contrast, a study of Austrian patients with high-grade CIN lesions demonstrated no significant association with the* IL1RN* VNTR polymorphic site [78]. Unfortunately, no data on HPV type distribution was reported in the Austrian study, rendering assumptions as to the cause of this difference between populations difficult.

Interleukin-10 (IL-10) has both immunosuppressive and antiangiogenic effects and may therefore exert both tumour-promoting and antitumour effects [79, 80]. Associations with both increased and decreased IL-10 levels in cervical cancer have been shown in different studies [81]. The* IL10* c.-1082A>G polymorphism has been shown to influence levels of IL-10 production, with major allele homozygosity being associated with low IL-10 levels, heterozygosity with intermediate levels, and minor allele homozygosity with high levels [82]. Several studies have investigated this SNP in cervical carcinoma with varying and often contradictory results [23, 24, 26, 28, 83–86]. A recent meta-analysis with pooled data from 1498 cases and 1608 controls showed no significant association of this SNP with cervical carcinoma risk [25]. However, the same meta-analysis did find a significant association for another* IL10* promotor SNP (c.-592C>A), with occurrence of the minor allele associated with increased cervical cancer risk, especially among Asian patients [25]. This association was also found in Mexican, Dutch, and Indian studies, as well as a more recent meta-analysis [16, 26–28]. The seemingly contradictory effects of IL-10 on carcinogenesis might have different implications for HPV infection and cervical cancer, given that angiogenesis is likely to be more important in later development of malignant tumours than in viral infection and persistence [87]. It is therefore possible that the heterogeneous effect of* IL10* polymorphisms in HPV and cervical cancer might be tied to the stage of lesions.

### 3.3. Chemokines

To date, polymorphisms in only one chemokine gene,* CXCL12,* have been reported in relation to cervical carcinoma. Chemokine CXCL12, also known as stromal-cell derived factor 1 (SDF-1), directs leucocyte migration and, through interactions with its receptor CXCR4 [88], it is also involved in the regulation of metastatic behaviour of certain tumour cell lines [89, 90]. One North American study investigated the role of several* CXCL12* SNPs in cervical cancer and identified one intronic SNP (rs266085), where the minor allele was associated with a decreased risk of cervical cancer. Using haplotype interaction analysis, this group identified a combination of three SNPs (rs266085, rs266093, and rs17885289), which was associated with cervical cancer risk [29]. The location of these SNPs in the 5′ UTR (rs17885289), second intron (rs266085), and 3′ UTR (rs266093) regions of the* CXCL12* gene is consistent with several possible aetiologic mechanisms for the identified association with cervical carcinoma, including alternative splicing and regulation of CXCL12 induction [91–93]. In contrast to this study, a separate study among Han Chinese women demonstrated a significant association of the rs266085 minor allele with higher risk of cervical cancer [30]. Stronger CXCL12 induction may have similar consequences as those seen in patients with mutations in the* CXCR4* gene, which lead to increased cellular responsiveness to CXCL12, yielding a rare syndrome characterised by immune deficits and extensive HPV-induced lesions [94, 95].

## 4. Receptors

Receptors bind a wide variety of ligands, including cytokines, costimulation transmembrane proteins, and pathogen-associated molecular patterns (PAMPs). Due to the different ligands and functions associated with them, many different receptors have been found to be associated with antitumour immunity. Genetic variation in the corresponding genes has been found to be strongly associated with HPV infection and subsequent cervical carcinoma.

The cell-surface FAS receptor induces apoptosis after binding by the FAS ligand (FASL) [96]. Two SNPs in the promotor region of the* FAS* gene have been identified: c.-1377G>A and c.-670G>A (Table 4). These SNPs disrupt Sp1 and STAT1 transcription factor binding sites, thus diminishing promotor activity and leading to FAS downregulation. Both SNPs are associated with several diseases, including acute myeloid leukaemia and systemic lupus erythematosus [97, 98]. Polymorphism at c.-670G>A has been found to be associated with cervical carcinogenesis, with the minor allele associated with higher risk of high-grade CIN and cervical cancer [31, 32]. Other studies have shown varying associations of* FAS* c.-670G>A with cervical carcinogenesis. A recent meta-analysis showed no significant association of this SNP with cervical cancer either in the overall population or in ethnical subgroups [33]. The c.-1377A>G SNP does not seem to be associated with cervical neoplasia individually [35] but has been shown to strengthen the effect of c.-670G>A [31].

One SNP in the* FASL* gene has been investigated for association with cervical cancer (Table 4).* FASL* c.-844T>C lies within a putative binding motif for CAAT/enhancer-binding protein (C/EBP*β*) and the two resulting alleles have different affinities for C/EBP*β*. This SNP has been demonstrated to have functional consequences: minor allele homozygosity has been linked to increased FASL expression and alteration of FASL-mediated signalling in lymphocytes [99]. Consequently, one study has demonstrated an association with cervical carcinoma risk [34]. However, in a recent meta-analysis, the* FASL* c.-844T>C SNP was not associated with cervical carcinoma risk [35].

### 4.1. Toll-Like Receptors (Table 5)

Toll-like receptors (TLRs) are transmembrane proteins that recognise pathogen-associated molecular patterns (PAMPs), the conserved structural motifs in pathogenic organisms [100]. TLRs are normally anchored in the plasma membrane but can also be present in intracellular membrane compartments, such as endosomes or lysosomes [100].

Toll-like receptor 2 (TLR2) is a pattern recognition receptor that senses the presence of bacterial lipoproteins and other components of bacteria and fungi [101]. However, there is mounting evidence that points to its putative role in sensing viral pathogens as well [102, 103]. Accordingly, the* TLR2* c.+613T>C SNP showed association with cervical cancer in a Costa Rican population [36].

TLR4 plays an important role in recognising lipopolysaccharide molecules present in Gram-negative bacteria; however, as in the case of TLR2, recognition of viruses has also been implicated [104]. Heterozygosity at* TLR4* c.+936C>T polymorphism was associated with stage II cervical cancer in a North Indian population [37].

Toll-like receptor 9 (TLR9) recognises nonmethylated CpG DNA sequences, ubiquitous in bacterial and viral genomes, for instance, HPV16. Viral oncoproteins E6 and E7 block the expression of this receptor, thereby contributing to HPV immune evasion [105].* TLR9* polymorphisms may therefore influence HPV persistence and cervical carcinogenesis. The* TLR9* c.+2848G>A SNP was shown to be associated with risk of cervical carcinoma, with the minor allele associated with an increased cancer risk among Chinese Han women [38]. Similar results were obtained in a large Polish study [39]. Similarly, the* TLR9* c.-1486T>C SNP has been associated with cervical cancer risk in various studies [39, 40].

## 5. Antigen-Processing Machinery

The antigen-processing machinery (APM) is responsible for the generation, trimming, transport, and loading of peptides derived from intracellular proteins to be presented by molecules of the human leucocyte antigen (HLA) family to cytotoxic T lymphocytes [106]. The main components of the APM are low molecular-weight proteins (LMP) 2 and 7, involved in generation of peptides from intracellular proteins, transporter associated with antigen presentations (TAP) 1 and 2, involved in transporting peptides from the cytosol to the endoplasmic reticulum, where peptides undergo further trimming by endoplasmic reticulum aminopeptidase associated with antigen presentations (ERAP) 1 and 2, and chaperone molecules including tapasin, calnexin, calreticulin, and Erp57, which are responsible for loading of the peptides onto empty HLA class 1 molecules. As both viral infection and malignant transformation lead to the occurrence of aberrant intracellular proteins, the APM is hypothesised to be an important mechanism of recognition and lysis of virally infected and neoplastic transformed cells. Correspondingly, polymorphisms in various genes encoding APM components have demonstrated association with increased cervical carcinoma risk as well as with worse clinical outcome (Table 6). The most probable explanation for this is that genetic variation in certain APM components may ultimately lead to alterations in the immunogenicity of the repertoire of peptides presented by HLA class 1 molecules. In particular, SNPs in the* LMP7*,* TAP2,* and* ERAP1* have been found to be associated with cervical carcinoma risk, lymph node metastases, and overall survival, both individually and in specific haplotype configurations [41, 45]. Though similar gene and haplotype associations have been found in several populations, including Dutch, Indonesian, Austrian Caucasian, North Indian, and North American, the actual genes and gene combinations involved differ greatly between the various populations [42–44, 107].

## 6. Discussion

There is a great diversity of immunogenetic associations with HPV infection, persistence, and cervical neoplastic transformation, based on the overwhelming body of continuously growing data. Contradicting results indicated by different studies attest to the complexity of the topic. Moreover, the wide variation in sample size amongst the various studies can lead to underestimation of the actual strength of association of particular loci with disease [108]. Multiple gene-gene and gene-environment interactions ought to be inspected on par, in order to gain a comprehensive, systematic insight into the molecular network underlying the aetiology of HPV persistence and cervical cancer. However, as is the case with low-penetrance alleles, we can only speak in terms of attributable risks. For a considerable number of genes listed in this review, contributions of each polymorphism or even a haplotype to a disease are often modest. Just as HPV infection alone is not sufficient to develop cervical dysplasia or cervical carcinoma, one particular genotype or haplotype is not likely to individually cause disease. A possibly more plausible scenario is that in which, following infection with an oncogenic HPV type and initial malignant transformation of cervical epithelial cells, certain SNPs in APM components may lead to a less immunogenic peptide repertoire to be presented to local immune cells. The presentation of these peptides may be further influenced by polymorphisms in HLA genes. The resulting immune response is then further attenuated by underlying SNPs in cytokine genes and receptor/KIR genes, which lead to a less effective overall local immune response. The end result of all these factors may then be further development and progression of malignant cells, ultimately leading to high-stage cervical carcinoma. This hypothesis is based on an interplay between host genomic factors, environmental factors, and HPV-related factors, which may explain the sometimes contradictory associations found for genes among different populations.

An emerging field in genetic association studies is that of haplotype interaction analysis, which investigates the association of specific SNP combinations (often spanning multiple chromosomes) with particular diseases [109]. This is based on the assumption that where individual SNPs may exert a (weak) influence on disease traits, the combination of particular alleles at these loci may bear a stronger association. Until now, all such analyses have been limited to genes from the same category (e.g., haplotypes spanning only APM component genes). The logical next step would be to investigate combinations of a larger spectrum of gene variants (e.g., haplotypes spanning APM component genes, combined with cytokine and receptor genes).

The increasingly popular genome-wide association study (GWAS) approach for the purpose of identifying polymorphisms of interest appears promising for the purpose of elucidating the susceptibility to persistent HPV infection as well as progression of cervical neoplasia [53, 110]. Also, the emergence of integrative sciences such as systems biology and immunoinformatics appears to be an encouraging approach that could help explain these interactions and provide a full understanding of the aetiology of these diseases [111, 112]. Understanding the immunogenetic networks that underlie complex diseases such as cancer would hopefully bring personalised prevention, diagnosis, and treatment closer to their successful implementation into the healthcare system. It would be interesting to see whether this can be accomplished by incorporating these factors with other biomarkers most predictive of cervical lesions,* MAL/CADM1* methylation pattern, p16INK-4a/Ki-67 dual immunostaining, and viral integration [113]. These markers appear most promising for the usage in successful triage of hrHPV-positive women for the purpose of successful screening for high-grade lesions. Aside from the potential to alter diagnostic risk assessment, detailed insights into the roles of immunogenetic factors in HPV infection and cervical cancer can contribute to other levels of prevention as well as therapeutics. Combining knowledge of a person's HPV status with immunogenetic factors (both in the genes described in this review and in other immunologically important genes, e.g., HLA) could enable the development of host-directed treatment based on a person's immunogenetic profile, which may lead to a more effective cure or remission with minimised side effects. Developing a therapeutic HPV vaccine would provide novel means of treatment for individuals already infected with hrHPV or suffering from related diseases [114]. Elucidating the precise role of immunogenetics in HPV infection and cervical neoplasia is a prerequisite for making these advances.

Ultimately, by combining data regarding HPV infection and distribution with host genomic data, it may be possible to make individual “predictions” not just of the risk of developing cervical cancer and its progression but also of the efficacy of therapies and, equally important, the efficacy of anti-HPV vaccination programmes, all of which will ultimately facilitate the development of tailor-made, personalised interventions.

## Figures and Tables

**Table 1:** Overview of polymorphisms in genes encoding proinflammatory cytokines and association with cervical neoplasia risk.

Gene	Polymorphism^a^	Cohort	Cases (*n*)	Population	Distribution	Risk (OR, 95% CI); PAF^b,c,d^	Reference
*IL1B*	c.-511C>T (rs16944)	Cervical cancer	182	Korean	Genotypes	**CT: 2.83 (1.52**–**5.28); 44.8%** TT: 1.68 (0.85–3.32) **CT/TT: 2.42 (1.31**–**4.46); 54.2%**	[12]
			150	North Indian	Alleles	**T: 1.83 (1.28**–**2.61); 33.9%**	[13]
					Genotypes	CT: 1.37 (0.59–3.20) **TT: 2.77 (1.21**–**6.41); 36.6%**	
			404	Chinese Han	Genotypes	**CT: 1.53 (1.09**–**2.15); 19.5%** TT: 1.47 (0.97–2.24) **CT/TT: 1.52 (1.10**–**2.09); 26.2%**	[14]
			100	Egyptian	Alleles	**T: 2.00 (1.19**–**3.38); 37.5%**	[15]
					Genotypes	CT: 0.72 (0.36–1.43) **TT: 2.16 (1.07**–**4.33); 30.6%** **CT/TT: 2.91 (1.01**–**8.36); 61.0%**	
			736	Asian	Genotypes	**CT: 1.69 (1.29**–**2.22)** **TT: 1.64 (1.19**–**2.25)** **CT/TT: 1.69 (1.30**–**2.18)**	[16]

*TNFA*	c.-308G>A (rs1800629)	Cervical cancer	195	North Portuguese	Genotypes	**GA: 1.81 (1.10**–**2.97); 11.5%** AA: 2.54 (0.65–10.52) **GA/AA: 1.88 (1.20**–**2.94); 13.7%**	[17]
		Cervical cancer	2279	Varied	Alleles	**A: 1.57 (1.21**–**2.04)**	[16]
		Cervical cancer + CIN	165	Indo-Aryan	Alleles	**A: 2.70 (1.41**–**5.15); 13.2%**	[18]

*IFNG*	c.+874T>A (rs2430561)	Cervical cancer	200	North Indian	Genotypes	**TA: 3.30 (2.05**–**5.20); 43.6%** AA: 1.90 (0.90–3.90) **TA/AA: 2.90 (1.90**–**4.60); 48.8%**	[19]
			200	North Indian	Alleles	**A: 1.54 (1.17**–**2.03); 23.0%**	[20]
					Genotypes	TA: 1.56 (0.88–2.78) **AA: 2.43 (1.34**–**4.42); 25.3%**	
			186	Chinese	Alleles	**A: 1.47 (1.10**–**1.97); 17.6%**	[21]
					Genotypes	TA: 1.58 (0.86–2.91) **AA: 2.22 (1.19**–**4.15); 24.0%**	
			1116	Varied	Genotypes	**TA/AA: 1.399 (1.097**–**1.784)**	[22]
			1532	Varied	Alleles	**A: 1.30 (1.01**–**1.69)**	[16]

*n*: number of cases; OR: odds ratio; 95% CI: 95% confidence interval; CIN: cervical intraepithelial neoplasia; PAF: population attributable fraction.

^a^Nucleotide variation and dbSNP reference number.

^b^OR relative to major allele or major allele homozygotes.

^c^PAF listed if OR > 1.00.

^d^Significant associations listed in bold.

**Table 2:** Overview of polymorphisms in genes encoding anti-inflammatory cytokines and association with cervical neoplasia risk.

Gene	Polymorphism^a^	Cohort	Cases (*n*)	Population	Distribution	Risk (OR, 95% CI); PAF^b,c,d^	Reference
*IL1RN*	VNTR alleles 1–5	Cervical cancer	150	North Indian	Alleles	**2: 2.33 (1.57**–**3.44); 19.4%** 3: 0.89 (0.27–2.77) 4: 1.00 (0.18–5.33)	[13]
					Haplotypes *(ILRN-VNTR*/*IL1B c.-511C>T)*	**1/T: 1.71 (1.12**–**2.62); 18.7%** 2/C: 1.98 (0.95–4.14) **2/T: 4.08 (2.38**–**7.04); 20.1%** 3/T: 1.26 (0.37–4.14) 4/T: 1.89 (0.29–2.23)	

*IL10*	c.-1082A>G (rs1800896)	CIN Cervical cancer	163 104	Japanese	Genotypes	CIN **AG/GG: 2.2 (1.2**–**4.2); 53.9% **Cervical cancer **AG/GG: 3.9 (2.1**–**7.3); 70.8%**	[23]
		Cervical cancer	77	African	Genotypes	**AG: 0.28 (0.12**–**0.61)**	[24]
		Cervical cancer	667	Caucasian	Alleles	**G: 0.39 (0.32**–**0.47)**	[25]
		Cervical cancer	256	Indian	Genotypes	**AG/GG: 3.67 (2.33**–**5.77); 52.2%**	[26]

*IL10*	c.-592C>A (rs1800872)	Cervical cancer	2396	Asian Caucasian	Alleles	**A: 1.16 (1.04**–**1.31)**	[25]
		Cervical cancer	2183	Varied	Alleles	**A: 1.17 (1.04**–**1.33)**	[16]
		Squamous intraepithelial cervical lesions	204	Mexican	Alleles	A: 1.32 (0.97–1.81)	[27]
					Genotypes	**CA/AA: 2.02(1.26**–**3.25); 38.4%**	
		CIN II/III Cervical cancer	263 667	Dutch	Genotypes	CIN **CA: 1.44 (1.06**–**1.97); 11.2%** AA: 1.09 (0.53–2.26) **CA/AA: 1.40 (1.04**–**1.88); 11.7%** Cervical cancer **CA: 1.36 (1.07**–**1.73); 9.3%** AA: 1.30 (0.73–2.29) **CA/AA: 1.34 (1.05**–**1.72); 10.1%**	[28]

*n*: number of cases; OR: odds ratio; 95% CI: 95% confidence interval; CIN: cervical intraepithelial neoplasia; PAF: population attributable fraction.

^a^Nucleotide variation and dbSNP reference number.

^b^OR relative to major allele or major allele homozygotes.

^c^PAF listed if OR > 1.00.

^d^Significant associations listed in bold.

**Table 3:** Overview of polymorphisms in gene encoding chemokines and association with cervical neoplasia risk.

Gene	Polymorphism^a^	Cohort	Cases (*n*)	Population	Distribution	Risk (OR, 95% CI); PAF^b,c,d^	Reference
*CXCL12 (SDF-1)*	G>A rs266085	Cervical cancer	917	American	Alleles	**A: 0.74 (0.56**–**0.98)**	[29]
					Haplotypes (rs266085/ rs17885289G>A/ rs266093C>G)	**A/A/C: 0.69 (0.54**–**0.89)**	
			348	Han Chinese	Alleles	**A: 1.41 (1.14**–**1.74); 17.1%**	[30]

*n*: number of cases; OR: odds ratio; 95% CI: 95% confidence interval.

^a^Nucleotide variation and dbSNP reference number.

^b^OR relative to major allele or major allele homozygotes.

^c^PAF listed if OR > 1.00.

^d^Significant associations listed in bold.

**Table 4:** Overview of polymorphism in *FAS* and *FASL* genes encoding receptors and association with cervical neoplasia risk.

Gene	Polymorphism^a^	Cohort	Cases (*n*)	Population	Distribution	Risk (OR, 95% CI); PAF^b,c,d^	Reference
*FAS*	c.-670G>A (rs1800682)	CIN Cervical cancer	143 175	Han Chinese	Alleles	**A: 1.26 (1.01**–**1.57); 12.4%**	[31]
					Genotypes	GA: 1.11 (0.60–2.02) AA: 1.83 (0.97–3.44)	
					Haplotypes *(FAS c.-670/ c.-1377A>G)*	G/G: 1.38 (0.80–2.37) **A/A: 3.05 (1.28**–**7.30); 11.3%** A/G: 1.27 (1.00–1.60)	
		CIN I CIN II/III Cervical cancer	104 131 176	Taiwanese	Alleles	CIN I A: 1.1 (0.8–1.6) CIN II/III A: 1.1 (0.8–1.6) Cervical cancer **A: 1.5 (1.1**–**2.0); 20.5%**	[32]
		Cervical cancer	2317	Varied	Alleles	A: 0.97 (0.84–1.11)	[33]

*FASL*	c.-844T>C (rs736110)	Cervical cancer	314	Chinese	Genotypes	TC: 1.68 (0.78–3.66) **CC: 3.05 (1.43**–**6.52); 41.3%**	[34]
			2485	Varied	Alleles	C: 1.12 (0.91–1.36)	[35]

*n*: number of cases; OR: odds ratio; 95% CI: 95% confidence interval; CIN: cervical intraepithelial neoplasia; PAF: population attributable fraction.

^a^Nucleotide variation and dbSNP reference number.

^b^OR relative to major allele or major allele homozygotes.

^c^PAF listed if OR > 1.00.

^d^Significant associations listed in bold.

**Table 5:** Overview of polymorphisms in toll-like receptor genes encoding receptors and association with cervical neoplasia risk.

Gene	Polymorphism^a^	Cohort	Cases (*n*)	Population	Distribution	Risk (OR, 95% CI); PAF^b,c,d^	Reference
*TLR2*	c.+613T>C (rs3804100)	CIN III Cervical cancer	470	Costa Rican	Genotypes	**TC: 0.61 (0.39**–**0.95)** CC: 0.56 (0.09–3.50) **TC/CC: 0.61 (0.40**–**0.93)**	[36]

*TLR4*	c.+936C>T (rs4986791)	Cervical cancer	150	North Indian	Genotypes	Overall CT: 1.50 (0.72–2.92) TT: 2.20 (0.20–24.76) Stage II **CT: 2.50 (1.03**–**6.12); 12.5%** TT: - Stage III CT: 1.30 (0.54–2.97) TT: 4.20 (0.37–47.64)	[37]

*TLR9*	c.+2848G>A (rs382140)	Cervical cancer	120	Chinese Han	Alleles	**A: 7.001 (2.422**–**20.23); 10.7%**	[38]
					Genotypes	**GA: 6.929 (1.534**–**33.30); 10.0%** **AA: 7.918 (1.797**–**64.52); 5.9%** **GA/AA: 7.259 (2.104**–**25.05); 15.8%**	
			426	Polish	Genotypes	**GA: 1.443 (1.019**–**2.043); 16.6%** **AA: 1.237 (1.016**–**1.507); 5.0%** GA/AA: 1.345 (0.976–1.855)	[39]

*TLR9*	c.-1486T>C (rs187084)	Cervical cancer	712	Chinese	Genotypes	**TC: 1.28 (1.01**–**1.62)** **TC/CC: 1.24 (1.01**–**1.53)**	[40]
			426	Polish	Genotypes	**TC: 1.371 (1.021**–**1.842); 13.0%** **CC: 1.300 (1.016**–**1.507); 4.4%** **TC/CC: 1.448 (1.099**–**1.908); 20.7%**	[39]

*n*: number of cases; OR: odds ratio; 95% CI: 95% confidence interval; CIN: cervical intraepithelial neoplasia; PAF: population attributable fraction.

^a^Nucleotide variation and dbSNP reference number.

^b^OR relative to major allele or major allele homozygotes.

^c^PAF listed if OR > 1.00.

^d^Significant associations listed in bold.

**Table 6:** Overview of polymorphisms in genes encoding antigen processing machinery components and association with cervical neoplasia risk/survival.

Gene	Polymorphism^a^	Cohort	Cases (*n*)	Population	Distribution	Risk (OR, 95% CI); PAF^b,c,d^	Reference
*ERAP1*	*ERAP1*-127 (c.+380G>C rs26653)	Cervical cancer	127	Dutch	Alleles	**C: 1.652 (1.106**–**2.467); 12.4%**	[41]
			98	Javanese	Alleles	**C: 0.656 (0.437**–**0.985)**	[42]
	*ERAP1*-730 (c.+2188C>G; rs27044)	Cervical cancer	127	Dutch	Alleles	**G: 1.685 (1.136**–**2.500); 13.6%**	[41]
			98	Javanese	Alleles	**G: 0.644 (0.431**–**0.962)**	[42]
	*ERAP1*-528 (c.+1583C>T; rs30187)	Cervical cancer	98	Javanese	Alleles	**T: 0.643 (0.429**–**0.964)**	[42]

*LMP7*	*LMP7*-145 (c.145C>A; rs2071543)	Cervical cancer	127	Dutch	Alleles	**A: 0.565 (0.346**–**0.920)**	[41]

*TAP1*	*TAP1*-333 (c.+1177A>G; rs4148880)	CIN III	114	American	Genotypes	**AG/GG: 0.28 (0.10**–**0.80)**	[43]

*TAP1*	*TAP1*-637 (A>G; rs1135216)	CIN III	114	American	Genotypes	**AG/GG: 0.27 (0.1**–**0.7)**	[43]

*TAP2*	*TAP2*-651 (c.+1951C>A; rs4148876)	Cervical cancer	127	Dutch	Alleles	**A: 0.481 (0.246**–**0.942)**	[41]
			103	Balinese	Alleles	**A: 3.837 (2.379**–**6.189); 47.2%**	[42]

*TAP2*	*TAP2*-379 (c.1135G>A; rs4148873)	CIN	616	Caucasian	Alleles	**A: 0.5 (0.4**–**0.8)**	[44]

*Various APM gene combinations*	*ERAP1*-127(G>C)/ *ERAP1*-730(C>G)/ *TAP2*-651(C>A)/ *LMP7*-145(C>A)	Cervical cancer	127	Dutch	Haplotypes	**C/G/C/C: 3.024 (1.656**–**5.519); 11.6%**	[41]
	*ERAP1*-575(C>T)/ *TAP2*-379(G>A)/ *TAP2*-651(C>A)	Cervical cancer	98	Javanese	Haplotypes	**T/C/G: 3.36 (0.98**–**11.56); 3.9%**	[42]
	*ERAP1*-56(G>A)/ *ERAP1*-127 (G>C)	Cervical cancer survival	75	Dutch	Haplotypes	**G/C heterozygotes: 0.219 (0.065**–**0.731**)^e^	[45]

*n*: number of cases; OR: odds ratio; 95% CI: 95% confidence interval; CIN: cervical intraepithelial neoplasia; PAF: population attributable fraction.

^a^Nucleotide variation and dbSNP reference number.

^b^OR relative to major allele or major allele homozygotes.

^c^PAF listed if OR > 1.00.

^d^Significant associations listed in bold.

^e^Hazard ratio listed for survival analysis.
